# happi: a hierarchical approach to pangenomics inference

**DOI:** 10.1186/s13059-023-03040-6

**Published:** 2023-09-29

**Authors:** Pauline Trinh, David S. Clausen, Amy D. Willis

**Affiliations:** 1 Department of Environmental & Occupational Health Sciences, University of Washington, Seattle, WA USA; 2 Department of Biostatistics, University of Washington, Seattle, WA USA

**Keywords:** Shotgun metagenomics, Metagenome-assembled genomes, Microbiome, Statistical models, Hypothesis testing

## Abstract

Recovering metagenome-assembled genomes (MAGs) from shotgun sequencing data is an increasingly common task in microbiome studies, as MAGs provide deeper insight into the functional potential of both culturable and non-culturable microorganisms. However, metagenome-assembled genomes vary in quality and may contain omissions and contamination. These errors present challenges for detecting genes and comparing gene enrichment across sample types. To address this, we propose happi, an approach to testing hypotheses about gene enrichment that accounts for genome quality. We illustrate the advantages of happi over existing approaches using published Saccharibacteria MAGs, *Streptococcus thermophilus* MAGs, and via simulation.

**Supplementary Information:**

The online version contains supplementary material available at 10.1186/s13059-023-03040-6.

## Background

Members of the same bacterial species can display a wide variety of different phenotypes, and intra-species variation in pathogenicity, virulence, drug resistance, environmental range, and stress response has been observed across the tree of life [1–5]. Variation in phenotypes can in part be explained by genotypic variation, which is also considerable because mechanisms of genetic recombination in bacteria facilitate large genetic variation even within narrow organismal groups. For example, of 7385 gene clusters observed in a study of 31 genomes in the genus *Prochlorococcus*, only 766 gene clusters were detected in all genomes [6]. We refer to the set of genes shared by all members of a clade as the *core genome* and we refer to the set of genes not shared by all members as the *accessory genome* [7]. Together, these sets of genes comprise a clade’s *pangenome*: the entire collection of genes present in one or more organisms within the clade. In this paper, we describe a novel tool for pangenome analysis. Our tool is a statistical method to model the association between gene presence and covariates (predictors). Our method offers interpretable parameter estimates, a fast algorithm for estimation, and a flexible hypothesis testing procedure.

While cultivation-based studies have historically been used to study the gene content of bacteria, it has become increasingly common to employ shotgun metagenomics to study bacterial genomes and communities. Shotgun metagenomic sequencing involves untargeted sequencing of all DNA in an environment, enabling the study of genomes in their environmental context. Short reads from shotgun sequencing can be assembled into contigs and binned into metagenome-assembled genomes (MAGs), which represent a partial reconstruction of an individual bacterial genome. Despite major advances in methods for binning MAGs, MAGs can contain two types of errors. First, there can be genes that are truly present in the genome the MAG represents but are unobserved in a MAG. Common reasons for this error include inadequate sequencing depth, high diversity in the metagenomes under study, and the inherent limitations of short read sequencing for reconstructing repetitive regions [8–12]. A second type of error in MAGs is erroneously observed genes: genes that are included in a MAG that are not truly present in the originating genome. This phenomenon is often referred to as contamination. The use of automated binning tools in the absence of manual inspection and refinement can lead to elevated rates of contamination. For example, the identification of contaminating contigs from manual refinement of MAGs produced by a massive unsupervised genome reconstruction effort removed 30 putative functions from a single contaminated genome [13, 14].

To address the challenges that contaminating and unobserved genes create for detecting enriched genes, our proposed method incorporates information about each genome’s quality. Under our proposed model, a gene may be unobserved in a genome either because the gene is not present in the source genome or because it could not be recovered from the obtained sequencing data. Our proposed method is based on the rationale that poorer quality (i.e., more shallowly sequenced) genomes or metagenomes are more likely to fail to detect genes. If, for example, the coverage of short reads across the genome was high and most of the expected core genes were observed, then the lack of detection of a given gene is more likely attributable to its true absence. The user can select which variables they believe to be the most informative for genome quality in their dataset. We develop estimators of the parameters of our model, discuss interpretation of model parameters, propose a hypothesis testing approach, and illustrate the performance of our model on shotgun sequencing and simulated data.

## Results

### A hierarchical model for gene presence

We present a hierarchical model for the association between bacterial gene presence and covariates of interest (e.g., host treatment status, environment of origin, relevant confounders, etc.). We consider observations on *n* genomes, which could be either metagenome-assembled genomes, isolate genomes, reference genomes, or any combination. Let $Y_{i}$ be an indicator variable for the gene of interest being *observed* in genome *i*, $Y_{i} = 1$ if the gene is observed in genome *i* and $Y_{i} = 0$ otherwise. However, we are not interested in whether the gene is *observed* in each genome — we are interested in whether it is *present* in each genome. To this end, we define $\lambda _{i}$ to be a latent (unobserved) random variable that indicates if the gene is truly present in genome *i* ($\lambda _i = 1$ if present).

We propose a logistic model to connect gene presence to covariate vector $X_i \in \mathbb {R}^p$:

1 $$\begin{aligned} \log \left( \frac{Pr(\lambda _{i}=1|X_{i})}{Pr(\lambda _{i}=0|X_{i})}\right) = X_{i}^T \beta , \end{aligned}$$

where the $\lambda _i$s are conditionally independent given $X_i$ and follow a Bernoulli distribution. Therefore, when comparing groups of genomes that differ by one unit in $X_{\cdot k}$ but are alike with respect to $X_{\cdot 1}, X_{\cdot 2}, \ldots , X_{\cdot ,k-1}, X_{\cdot ,k+1}, \ldots , X_{\cdot p}$, $\beta _k$ gives the difference in the log-odds that the gene will be present between these two groups of genomes. To connect $\lambda _{i}$ to $Y_{i}$ we propose the following model

2 $$\begin{aligned} Pr(Y_{i}=1| \lambda _{i}=\ell , M_{i}) = \left\{ \begin{array}{ll} f(M_{i})\qquad \qquad &{}\ell = 1 \\ \varepsilon &{}\ell = 0, \end{array}\right. \end{aligned}$$

where $Y_{i}$ are conditionally independent Bernoulli distribution random variables; $\varepsilon$ is the probability that a gene is observed in a genome in which it is absent (e.g., due to contamination or crosstalk); $M_{i} \in \mathbb {R}^{q}$ is a vector of genome quality covariates; and $f(\cdot ) :\mathbb {R}^{q} \rightarrow \mathbb {R}$ is a flexible function to connect quality variables to the probability of detecting a present gene. Relevant quality variables are context-dependent and could include coverage of the gene from metagenomic read recruitment, completion (percentage of single copy core genes observed in the genome), redundancy (percentage of single copy core genes observed more than once in the genome), and an indicator for the genome originating from an isolated bacterial population.

### Parameter estimation

The latent variable structure of our model makes the expectation-maximization algorithm [15] an appealing choice for estimating unknown parameters $\theta = \left( \beta , f\right)$. Because we do not observe $\{\lambda _{i}\}_{i=1}^n$, $\varepsilon$ and *f* are not, in general, jointly identifiable. Therefore, we treat $\varepsilon$ as a hyperparameter that can be fixed by the user or leveraged for sensitivity analyses. To improve stability of parameter estimates, we impose a Firth-type penalty on $\beta$. The complete data penalized log-likelihood is linear in $\lambda _i$, which allows us to simplify the expected complete data penalized log-likelihood at step *t* of an EM iteration as

3 $$\begin{aligned} \mathbb {E}_{\varvec{\lambda }|\textbf{Y}, \theta ^{(t - 1)}} \big [l(\beta , \tilde{f}, \tilde{\varepsilon }| \textbf{Y}, \varvec{\lambda })\big ]= & {} \sum _{i = 1}^n \Bigg (p_i^{(t)}\left[ Y_i\tilde{f}(M_i) - \text {log}(1 + \text {exp}(\tilde{f}(M_i)))\right] \nonumber \\{} & {} \quad + (1 - p_i^{(t)})[Y_i\tilde{\varepsilon }- \text {log}\left( 1 + \text {exp}(\tilde{\varepsilon })\right) ]\nonumber \\{} & {} \quad + \left[ p_i^{(t)}X_i^T\beta - \text {log}\left( 1 + \text {exp}\left( X_i^T\beta \right) \right) \right] \Bigg )\nonumber \\{} & {} \quad + \frac{1}{2}\text {log}\left| \sum _{i=1}^n X_i X_i^{T}\frac{\exp \left( X_{i}^{T}\beta \right) }{1 + \exp \left( X_{i}^{T}\beta \right) }\left( 1-\text {expit}(X_{i}^T\beta )\right) \right| , \end{aligned}$$

where $\tilde{\varepsilon } = \text {logit}(\varepsilon )$, $\tilde{f}(x) = \text {logit}(f(x))$ for all *x*, and $p_i^{(t)} = \mathbb {E}[\lambda _i|Y_i, \theta ^{(t - 1)}]$ can be simplified as

4 $$\begin{aligned} p_i^{(t)} = \frac{Pr\left( Y_i |\lambda _i = 1,\theta ^{(t - 1)}\right) Pr\left( \lambda _i = 1| \theta ^{(t - 1)}\right) }{Pr\left( Y_i|\theta ^{(t - 1)}\right) }, \end{aligned}$$

where the terms in the numerator are given in (1) and (2), and the denominator is given by

5 $$\begin{aligned} Pr\left( Y_i|\theta ^{(t - 1)}\right)= & {} Pr(Y_i| \lambda _i = 1,\theta ^{(t - 1)}) Pr(\lambda _i = 1|\theta ^{(t - 1)})\nonumber \\{} & {} \quad + Pr(Y_i| \lambda _i = 0,\theta ^{(t - 1)}) Pr(\lambda _i = 0|\theta ^{(t - 1)}). \end{aligned}$$

We maximize the expected complete data penalized log-likelihood separately for $\beta$ and *f*. Owing to the form of the expected complete data penalized log-likelihood, efficient algorithms exist to perform each of these maximizations. Optimizing (3) with respect to $\beta$ is equivalent to fitting a binomial generalized linear model with logit link function for outcomes $p_{i}^{(t)}$ via Firth-penalized maximum likelihood, and we find Newton’s method to be stable and fast for this purpose.

Optimizing for *f* depends on the class of functions in which *f* falls. We investigated two flexible non-parametric options for *f*: $f \in \mathcal {F}$, where $\mathcal {F}$ is the class of bounded non-decreasing functions that map from $\mathbb {R}$ to $\mathbb {R}$, and $f \in \mathcal {I}$ where $\mathcal {I}$ is the class of linear combinations of *k* I-spline basis functions and a constant function where all basis functions have nonnegative coefficients. Both $f \in \mathcal {F}$ and $f \in \mathcal {I}$ result in a monotone estimate for *f*. To obtain the EM update for $f \in \mathcal {F}$, we use the primal active set algorithm of isotone [16] with custom loss function given by the first term in (3) plus a penalty term $-cosh\left( \left( \frac{m}{a}\right) ^2\right)$ to prevent $\left| \tilde{f}\right|$ from growing without bound. We found that setting $a=50$ gives a sensible tradeoff between algorithm convergence and numerical stability. To obtain the EM update for $f \in \mathcal {I}$, we fit a logistic regression on $p_{i}^{(t)}$ with predictors consisting of an I-spline basis with all non-intercept coefficients constrained to be nonnegative. We use the I-spline basis functions implemented in splines2 [17]. In an analysis where we used short-read subsampling to approximate an empirical *f*, we found that $f \in \mathcal {I}$ outperformed $f \in \mathcal {F}$ (see the “Methods: simulation studies:Evaluating estimators for *f*” section), and for that reason, we consider $f \in \mathcal {I}$ throughout the remainder of this manuscript. We run the estimation algorithm for $t_{\text {max}}$ steps or until the relative increase in the log-likelihood is below threshold $\Delta$ for 5 consecutive steps.

### Hypothesis testing

To enable inference on the odds that a gene will be present in groups of genomes that differ in their covariate attributes, we construct a hypothesis test for null hypotheses of the form $\textbf{A}\beta =c$ for $\textbf{A} \in \mathbb {R}^{h \times p}$ and $c \in \mathbb {R}^{h}$ where $\text {rank}(\textbf{A}) = h$. This allows testing of null hypotheses including $\beta _k=0$ (the odds that the gene will be present are equal when comparing groups of genomes that differ in $X_{\cdot k}$ but are alike with respect to $X_{\cdot 1}, X_{\cdot 2}, \ldots , X_{\cdot ,k-1}, X_{\cdot ,k+1}, \ldots , X_{\cdot , p}$). We propose to use a likelihood ratio test for $\textbf{A}\beta =c$, rejecting $H_0$ at level $\alpha$ if $Q_{LRT} = 2[\mathcal {L}(\hat{\theta })-\mathcal {L}(\hat{\theta }_{0})]$ exceeds the upper $100\alpha \%$ quantile of a $\chi ^{2}_h$ distribution, where $\hat{\theta }$ is the maximum likelihood estimate of $\theta$; $\hat{\theta }_{0}$ is the maximum likelihood estimate of $\theta$ under the null hypothesis; and $\mathcal {L}$ is the log-likelihood function:

6 $$\begin{aligned} \mathcal {L}(\theta ) = \sum _{i:y=0}^n \log \left( \frac{1-\varepsilon + \left( 1-f(M_i)\right) \exp \left( X_{i}^{T}\beta \right) }{1 + \exp \left( X_{i}^{T}\beta \right) } \right) + \sum _{i:y=1}^{n} \log \left( \frac{\varepsilon + f(M_{i})\exp \left( X_{i}^{T}\beta \right) }{1 + \exp \left( X_{i}^{T}\beta \right) }\right) \end{aligned}$$

When *n* is large, we find that the distribution of $Q_{LRT}$ is well-approximated by the $\chi ^{2}_h$ distribution (the “Simulation study” section). We also provide a nonparametric permutation-based hypothesis test (Additional file 1: S1) that controls error rates for modest sample sizes.

### Data analysis: Saccharibacteria MAGs

We consider a publicly-available dataset of $n=43$ non-redundant Saccharibacteria (TM7) MAGs recovered from supragingival plaque ($n=27$) and tongue dorsum ($n=16$) samples of seven individuals from [18] (see the “Methods: Saccharibacteria MAGs” section for more information). The wide variation in mean coverage across the MAGs (1.07 – 26.35$\times$) makes this an appealing dataset on which to illustrate our quality variable-adjusting pangenomics method.

We consider methods that allow us to test the null hypothesis that the probability (equivalently, odds) that a gene is present in Saccharibacteria genomes are equal for tongue and plaque-associated genomes. The alternative hypothesis is that the probabilities differ. We compare our proposed method (happi: a hierarchical approach to pangenomics inference) with two competitors: a logistic regression model for $Y_i$ with a likelihood ratio test (GLM-LRT) and a logistic regression model for $Y_i$ with a Rao test (GLM-Rao). Note that these latter two methods test hypotheses about the odds that a gene is observed, while our proposed approach tests hypotheses about the odds that a gene is present, but we believe that results can be reasonably compared between these methods. We consider a single quality variable $M_i$ for our analysis with happi: mean coverage across genome *i*. Given $n=43$ we run happi’s nonparametric hypothesis testing approach with 1000 permutations (see “Simulation study” section). Our primary comparison is with GLM-Rao, which is the method currently implemented for pangenomics hypothesis testing in anvi’o [18]. We also note that the results from GLM-Rao and GLM-LRT are highly correlated, especially for larger *p*-values.

Different methods identified different differentially present genes. Out of 713 COG functions tested, happi identified 176 differentially present genes when controlling false discovery rate (FDR) at the 5% level; GLM-LRT identified 219 genes; and GLM-Rao identified 175 genes. Out of the 176 genes identified as differentially present by happi, all 176 genes were also identified by GLM-LRT as differentially present and 166 genes were identified by GLM-Rao as differentially present.

To investigate the biological plausibility of the results from each method, we assessed the number of core genes that were identified as differentially present. [18] identified 172 COG functions in the TM7 core genome, and because core genes are genes that are present in most genomes of a particular clade, we consider differentially present core genes to be false positives. Controlling FDR at 5%, happi identified 6 out of 172 core genes to be differentially present; GLM-LRT identified 10 genes; and GLM-Rao identified 7 genes. While this difference is not substantial, we consider this reduction in the number of false positives to be an advantage of happi.

**Fig. 1:**
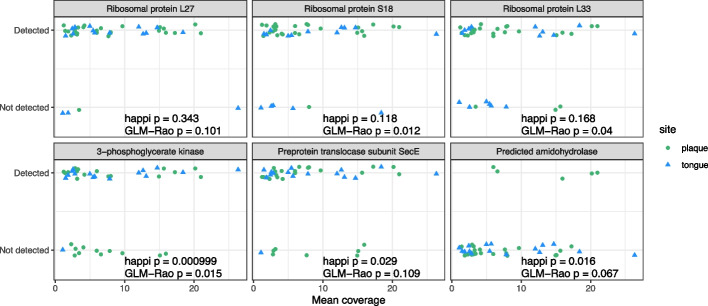
We test the null hypothesis that the probability that a gene is present are equal for tongue and plaque-associated Saccharibacteria genomes. The top 3 panels show core genes for which our proposed method resulted in greater *p*-values than existing methods, and the lower 3 panels show accessory genes for which our proposed method resulted in smaller *p*-values than existing methods. Our method reduced *p*-values when differences in detection cannot be attributed to genome quality factors (here, coverage), and increased *p*-values in situations when non-detection may be conflated with lower quality genomes. Points have been jittered vertically to separate observations

Our proposed method calculated lower *p*-values for 16% and 29% of genera compared to GLM-LRT and GLM-Rao. We show results from 6 specific model estimates in Fig. 1: 3 core genes for which happi produced greater *p*-values than GLM-Rao (upper panels; we believe these signals to be truly null), and 3 accessory genes for which it produced smaller *p*-values than GLM-Rao (lower panels). In all instances where happi produced greater *p*-values than GLM-Rao, non-detections generally occurred in genomes with low mean coverage. GLM-Rao does not account for coverage information, and so unlike happi, it can conflate gene absence with non-detections due to quality. We believe that statements about significance should be moderated when detection patterns can be attributable to quality variables and therefore that it is reasonable that *p*-values are larger in these three cases. In contrast, happi produced smaller *p*-values than GLM-Rao in instances when non-detections occurred for greater coverage MAGs, or broadly across the range of MAG coverage (lower panels). In these instances, differences in detection are less likely to be attributable to quality factors, and it is reasonable that the significance of findings can be strengthened by including data on quality variables.

### Data analysis: *Streptococcus thermophilus* MAGs

We also consider a larger data analysis of $n=157$ *Streptococcus thermophilus* MAGs available from MGnify [19]. These MAGs were recovered from human gastrointestinal samples from Spain ($n=82$) and Sweden ($n=75$), and test the null hypothesis that the probability that a gene is present in *S. thermophillus* genomes is equal for the Spanish and Swedish genomes. The CheckM completion and contamination [20] for these MAGs ranges between 51.62–99.89% and 0–4.89%, respectively. Given the large sample size, we use happi’s asymptotic hypothesis testing procedure. We consider CheckM genome completion as our quality variable $M_i$. Given the maximum contamination percentage across genomes in our sample, we choose $\varepsilon = 0.05$.

As in the “Data analysis: Saccharibacteria MAGs” section, different methods yielded different results. Out of 2799 genes tested, happi identified 219 differentially present genes when controlling FDR at the 5% level, GLM-LRT identified 311 genes, and GLM-Rao identified 254 genes. Out of the 219 genes identified as differentially present by happi, 202 genes were also identified as differentially present by GLM-LRT and 196 genes were identified as differentially present by GLM-Rao. To investigate the biological plausibility of the results, we assessed the number of core genes that were erroneously identified as differentially present by each method. Out of 813 core genes annotated by MGnify as belonging to the core *S. thermophilus* genome, happi identified 3 differentially present core genes when controlling FDR at the 5% level, GLM-LRT identified 27 core genes, and GLM-Rao identified 6 core genes. Only one (the nrdG gene) out of the 3 core genes that happi identified as differentially present was also identified as differentially present by GLM-LRT and GLM-Rao. Notably, happi identified the fewest number of differentially present core genes out of all methods investigated, which we view as evidence of an improved false positive rate in practice.

We also investigated the sensitivity of the results of happi to different choices of $\varepsilon$, the probability of observing a gene given that it is truly absent (Additional file 1: S2). For specific genes of interest, we encourage users to investigate plausible levels of $\varepsilon$ to confirm the robustness of their results to this hyperparameter. In general, we recommend choosing $\varepsilon$ based on genome redundancy metrices or based on other tuning parameters for MAG construction. We discourage further exploration of genes whose significantly differential presence hinges on the assumption of low genome contamination levels and is not robust across small increases in $\varepsilon$.

### Simulation study

Finally, we investigate the performance of our approach by evaluating its type 1 error rate and power. To generate data that most realistically reflects the relationship between coverage and gene detection in shotgun metagenomics studies, we construct $f(\cdot )$ for use in this simulation by subsampling short-reads from host-associated *E. coli* genomes ([21]; see “Methods: simulation studies” section). By utilizing an empirically constructed $f(\cdot )$ as the basis of our simulation study, we are able to simulate a relationship between coverage and genome quality that we believe is representative of many shotgun metagenomics studies. We consider $q=1$ and $q=2$, and let $M_i = 10 + 30\frac{i-1}{n-1}$, $X_{i1} = 1$, $X_{i2} = \mathcal {N}(\frac{i-1}{n-1}$, $\sigma = \sigma _x)$ and $\varepsilon =0$. $\sigma _x$ is a parameter that controls the degree of correlation between $M_{i}$ and $X_{i2}$, with larger values resulting in less correlation between quality variables and the predictor of interest. We simulate data according to the model described in (1) and (2), with $\beta = (0,0)^T$ for type 1 error simulations and $\beta = (0, \beta _1)^T$ with $\beta _1 \ne 0$ for power simulations. We investigate two happi-based approaches to hypothesis testing: an asymptotic approach (happi-a) and a nonparametric approach (happi-np). The asymptotic approach considers the distribution of $Q_{LRT}$ as a $\chi ^{2}_h$ distribution (which is the case for large sample sizes), while the nonparametric approach uses permutations to construct a sampling distribution for $Q_{LRT}$ (see Algorithm 1, Additional file 1: S1). In this simulation, we ran 1000 permutations for happi-np. GLM-LRT and GLM-Rao produced highly similar *p*-values (mean squared difference $1.3 \times 10^{-5}$, correlation = 0.99996, $n_{sim}= 3000$), and therefore we only show results for GLM-Rao.

**Fig. 2:**
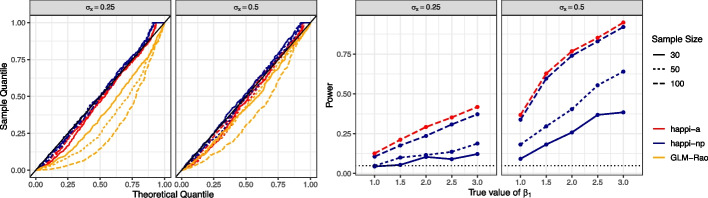
We investigate the performance of methods for testing for differential gene presence under simulation. (left) We find that logistic regression methods (e.g., GLM-Rao) do not control type 1 error, while happi-np controls type 1 error at nominal levels for all sample sizes. Additionally, we find that happi-a controls type 1 error for large sample sizes ($n=100$) and lower correlation between quality variables and the covariate of interest ($\sigma _x=0.5$). (right) For tests that control error rates at nominal levels, we evaluate the power of happi-np and happi-a to reject a false null hypothesis, finding that happi-a has slightly higher power than happi-np at sample size $n=100$. We find that power increases for all methods as sample sizes and effect sizes grow, but decreases with greater correlation between quality variables and the covariate of interest

The results of type 1 error rate simulations are shown in Fig. 2 (left panels). Notably, the logistic regression methods are anti-conservative and do not control type 1 error rates at nominal levels. For example, for a 5%-level test, type 1 error rates for GLM-LRT range from 8.8% ($n=30$ and $\sigma _x = 0.5$; 95% CI: 6.3–11.3%) to 32.2% ($n=100$ and $\sigma _x = 0.25$; 95% CI: 28.1–36.3%). Stated differently, under $H_0$, GLM-LRT will return *p*-values that are usually too small, leading to more frequent incorrect conclusions of an association. In contrast, happi-np does control the type 1 error rate, behaving near-exactly (viz., with nominal error rates). We estimate that happi-np’s type 1 error rates for a 5% test when $n=30$ and $\sigma _x = 0.5$ is 4.6% (95% CI: 2.7–6.4%), and when $n=100$ and $\sigma _x = 0.25$, happi-np’s empirical type 1 error rate is 6.8% (95% CI: 4.6–9.0%). Greater correlation between the quality variable (coverage) and the covariate of interest leads to greater anti-conservativeness for logistic regression methods, which incorrectly attribute differences in gene presence to the covariate of interest. However, happi-np appears to control type 1 error across the range of $\sigma _x$ investigated here. We further note that happi-a appears to control the type 1 error at larger sample sizes and lower correlation between the quality variable (coverage) and the covariate of interest with a type 1 error rate for a 5% test when $n=100$ and $\sigma _x = 0.5$ of 6.6% (95% CI: 4.4–8.8%). For even larger sample sizes, happi-a gives reliable inference (Additional file 1: Fig. S1) with the advantage of reduced run times compared to happi-np.

We show the power of happi-np and happi-a to correctly reject a null hypothesis at the 5% level in Fig. 2 (right panels). We do not evaluate power for GLM-Rao and GLM-LRT because they have uncontrolled type 1 error rates, making them invalid tests. Similarly, we do not evaluate the power for happi-a for all sample sizes, because it does not control type 1 error rates for $n=50$ and below. We observe that the power of happi-np to reject a false null hypothesis increases with the effect size and sample size but decreases with greater correlation between $M_i$ and $X_{i1}$. Stated differently, happi-np has low power to detect true associations between gene presence and covariates of interest when covariates are correlated with genome quality, though this can be remedied with larger sample sizes. Furthermore, we see that when $n=100$ and $\sigma _{x} = 0.5$, happi-a similarly has increased power with increasing effect size while maintaining slightly higher power than happi-np to reject a false null hypothesis.

Taken together, these results show that happi is robust to potential correlation between covariates of interest and genome quality. This is not the case for logistic regression-based methods, which cannot distinguish between differential gene presence due to genome quality and differential gene presence due to associations with covariates. No method will perform well under the alternative with small sample sizes and high correlation (see Fig. 2, third panel), but happi has some power for large sample sizes and large effect sizes in this setting and controls type 1 error at nominal levels regardless of the sample size. By using a simulation framework based on an empirically informed data generation structure, we demonstrate the disadvantages of using methods that do not account for differential genome quality. However, we note that in some settings (e.g., very deep short-read sequencing combined with short-read assembly) our assumption that the probability of gene detection increases with genome coverage may not hold [22]. We investigate the performance of happi under this form of model misspecification in Additional file 1: S3 and Fig. S2.

## Discussion

Many tools exist to study associations between microbial genome variation and microbial or host phenotypes [23–27]. Studies investigating the association between microbial genomes and phenotypes are often referred to as microbial genome-wide association studies (mGWAS) [28, 29]. Most mGWAS tools have been developed for the analysis of pure microbial isolates, and do not account for differential genome quality in genomes analyzed collectively. mGWAS tools may be better-suited when the hypothesized causal direction is that the presence of genetic features gives rise to a phenotypic characteristic, and not the reverse. In this paper, we propose and validate a novel method (happi) to understand how non-microbial variation (e.g., environmental variation) is associated with microbial genome variation. The implied direction of modeling is reversed in our model compared to mGWAS models: our response variable is gene presence rather than phenotype. This allows interrogation of questions about factors influencing selection pressures on genomes, rather than questions about the impact of the microbiome on phenotypic outcomes.

We view the main advantage of happi to be its use of data about genome quality factors in modeling gene presence to improve statistical inference. We believe this to be especially advantageous in the context of shotgun metagenomic data, where factors such as shallow sequencing depth may impact the ability to detect genes. To support the increasing use of shotgun metagenomic data to recover fragmented microbial genomes, researchers need methods that are capable of analyzing incomplete and imperfect genomes. While we are not aware of methods for modeling gene enrichment in MAGs, we offer comparisons to commonly used methods for analyzing near-complete genomes, such as logistic regression (used by anvi’o [18, 30]; see also [31]). In situations where differences in gene detection can be attributed to differences in genome quality, happi correctly infers that gene enrichment is ambiguous, and correspondingly identifies associations as less significant compared to competitor methods. However, in situations where genome quality cannot explain gene detection patterns, happi has greater precision than other methods and produces smaller *p*-values. We show via simulation that the advantages of happi are most pronounced when there is correlation between covariates and quality variables.

happi has reasonable run times on a modern laptop, averaging 2.11 s per gene over 500 genes in $n = 157$ samples with $t_{\text {max}} = 1000$ and $\Delta = 0.01$ on a 2.6 GHz i7 processor with 16 GB RAM with no parallelization. Since genes are treated independently, this analysis can be trivially parallelized, and furthermore, accuracy in estimation can be traded off for reduced runtime by reducing $t_{\text {max}}$ or increasing $\Delta$. happi’s nonparametric hypothesis testing procedure run with *P* permutations has runtime approximately *B* times greater than the asymptotic approximation. For our “Data analysis: Saccharibacteria MAGs” section analysis with $n = 43$, the average runtime was 4.25 minutes per gene with $B=1000$ for $t_{\text {max}} = 1000$, and $\Delta = 0.1$ on a 2.6 GHz i7 processor with 16 GB RAM and parallelized across 6 cores.

We suggest several avenues for further research. The first is to study the impact of experimental design on the statistical power of our proposed hypothesis testing procedure. Researchers often have to decide how to allocate budget across number of samples (including replicates and control data) and sequencing depth per sample. While existing guidelines for sequencing depth have focused on taxonomy estimation, MAG reconstruction, and gene detection [9–11, 32–34], our proposed modeling approach enables the principled study of the design of shotgun sequencing experiments to maximize power to detect differences in gene presence across sample groups.

We additionally note that the datasets and simulation study settings used in this paper to assess happi were low contamination ($<5\%$) MAGs. These MAGs reflect higher quality genomes that can be obtained using modern software for assembly, binning, and refinement [13]. Our supplementary investigation into the robustness of *p*-values obtained from running happi with varying levels of contamination (Additional file 1: Fig. S3) suggests that utilizing more highly contaminated MAGs would lead to larger overall *p*-values when using happi. However, specific recommendations on thresholds for the use of higher contamination levels in MAGs for reliable inference, as well as the incorporation of other genome quality metrics, require further research and development. This remains an ongoing area of investigation.

Our latent variable model also has possible utility for modeling the presence of amplicon sequence variants and could offer a method for studying patterns of sequence variant presence when shotgun sequencing is infeasible or not preferred. For example, if a sequence variant is observed $W_i$ times in sample *i*, then it would be reasonable to model $Y_i = \textbf{1}_{\{W_i > 0\}}$. This would permit inference on the equality of the probability that the sequence variant is absent in a sample across sample groups. Notably, by choosing an $\varepsilon > 0$ (e.g., via the use of negative control samples), happi can adjust for the impact of index switching in studies that leverage multiplexing [35, 36]. We leave the application of happi to modeling the presence of amplicon sequence variants to future research.

Collectively, we have shown that happi is accurate and robust, even when genome quality is correlated with gene presence predictors. As the recovery of metagenome-assembled genomes becomes increasingly common, statistical tools that account for errors in recovered genomes become increasingly necessary. By leveraging genome quality metrics to model gene presence, happi provides sensible and interpretable results in an analysis of metagenome-assembled genome data, improves statistical inference under simulation, and can run efficiently on a local machine. We view happi as a complementary tool to existing methods for the analysis of metagenomics data, such as methods for differential taxon abundance (e.g., MetaPhlAn [37]). Finally, by distributing open-source software in R implementing our proposed estimation and inference methods, we hope that happi can be used widely in a variety of genomics research settings. happi, along with workflows and vignettes demonstrating its use, is available as an open-source R package via https://github.com/statdivlab/happi under a BSD-3-Clause license.

## Conclusions

Fragmented microbial genomes, such as metagenome-assembled genomes, pose challenges in accurately detecting enriched genes due to the potential presence of contaminant genes or the possibility of missing genes altogether. To address these challenges, we present happi, a pangenomics method designed to test hypotheses about gene enrichment while taking into account genome quality. Using published shotgun sequencing data and simulations, we demonstrate the accuracy and robustness of happi to potential correlation between genome quality and covariates of interest. We also demonstrate a reduction in the number of false positives compared to existing methods. By leveraging genome quality metrics, happi improves statistical inference for gene enrichment hypotheses while providing sensible and easily interpretable results. To facilitate broad utilization and collaborative research in genomics, we distribute happi as documented, open-source software in R.

## Methods

### Methods: Saccharibacteria MAGs

The Saccharibacteria MAGs used in “Data analysis: Saccharibacteria MAGs” section, were taken from publicly available data [18]. Specifically, data on genome quality metrics (i.e., mean coverage) of these Saccharibacteria MAGs were retrieved from Supplementary materialshttps://doi.org/10.6084/m9.figshare.11634321 and information on the presence or absence of COG functions in each MAG was extracted from the Saccharibacteria pangenome contigs databases and profiles located at https://doi.org/10.6084/m9.figshare.12217811. Functional annotation of the genes was performed using NCBI’s Clusters of Orthologous Groups (COG) database [38]. Further details on sampling, assembly, binning, and refinement can be found in [18]. In our data analysis, we utilized happi’s nonparametric approach to hypothesis testing due to the limited sample size and specified $t_{\text {max}} = 1000$, $B = 1000$, $\Delta = 0.1$ and $\varepsilon = 0$. We set $\varepsilon = 0$ because these MAGs had undergone careful manual refinement to remove contamination from other genomes. We suggest the use of $\varepsilon > 0$ when binning is performed automatically and without additional manual refinement.

### Methods: *Streptococcus thermophilus*

The *Streptococcus thermophilus* MAGs used in the “Data analysis: *Streptococcus thermophilus* MAGs” section were taken from publicly available data [19]. Using the MGnify online tool, we queried for *Streptococcus thermophilus* MAGs from human gastrointestinal samples resulting in a match with MGYG000004345. Genome quality metrics, gene presence absence matrices, and annotated core genes data were retrieved from the pangenome analysis downloads tab of MGYG000004345 made available at [39]. The full metadata from MGnify studies can be found at [40] and were used to identify *Streptococcus thermophilus* MAGs from individuals originating from Sweden and Spain. Further details on the sampling, assembly, binning, and refinement of these genomes can be found in [19]. In our data analysis, we used happi’s asymptotic hypothesis testing approach and specified $t_{\text {max}} = 1000$, $\Delta = 0.01$ and $\varepsilon = 0.05$. We selected $\varepsilon = 0.05$ based on the maximum observed percent contamination across our genomes as determined by CheckM [20]. For our sensitivity analyses, we used various specifications of $\varepsilon = 0.01, 0.1$, $t_{\text {max}} = 1000$, and $\Delta = 0.01$.

### Methods: simulation studies

#### Subsampling study of *E. coli* isolate DRR102664

**Fig. 3:**
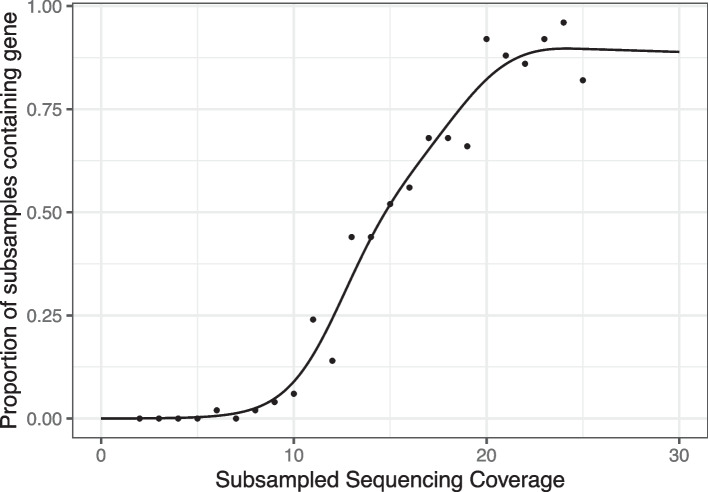
We subsampled reads from a publicly available *E. coli* isolate genome to understand the impact of coverage on the probability of detecting a gene, finding that the probability of detection increases with coverage. We use a nonparametric smoother to interpolate this curve and use it as the true function *f* in our simulations

To investigate the probability of detecting a gene that it is truly present ($Pr(Y_i=1|\lambda _i=1, M_i = m)$), we conducted a subsampling simulation study of an *E. coli* isolate genome taken from [21]. We selected *E. coli* isolate DRR102664 to perform our subsampling simulation and the eaeA gene (K12790) as our target gene of interest. In enteropathogenic Escherichia coli, the eaeA gene produces a 94-kDa outer membrane protein called intimin which has been shown to be necessary to produce the attaching-and-effacing lesion. For our subsampling study, we subsampled paired sequences 50 times from the DRR102664 genome at approximate coverages $m=(2\times , 3\times , ..., 24\times , 25\times )$. Coverages were estimated using the calculation $\frac{\text {read count} \times \text {read length}}{\text {genome length}}$. We annotated and identified the eaeA gene in each set of subsampled sequences and calculated the empirical probability of detection as the fraction of samples of coverage *m* that detected eaeA. The results of our subsampling investigation of the impact of coverage on the probability of detection given presence are shown in Fig. 3.

#### Evaluating estimators for *f*

Many different choices of functions *f* could be used to connect the probability of detecting a present gene to quality variables $M_i$. We evaluated two options under simulation: $f(M_i) \in \mathcal {F}$ for $\mathcal {F}$ the class of bounded non-decreasing functions and $f(M_i) \in \mathcal {I}$ for $\mathcal {I}$ the class of bounded non-decreasing functions. As in the “Simulation study” section, we set $M_i = 10 + 30\frac{i-1}{n-1}$, $X_{i1} = 1$, $X_{i2} = \mathcal {N}(\frac{i-1}{n-1}$, $\sigma = \sigma _x)$, $\beta _0 = 0$, and $\varepsilon =0$. The true $f(\cdot )$ in this simulation is a generalized additive model with binomial link function [41] fit to the observations shown in Fig. 3. This was done to select a true detection curve that well-reflects empirical probabilities of detecting a gene at a given coverage, such as gene eaeA in *E. coli* isolate genome DRR102664. We evaluated all estimators via mean squared error and median squared error for estimating $\beta _1$. We investigated all combinations of $n \in \{30, 50, 100\}$, $\beta _1 \in \{0.5, 1, 2\}$, and $\sigma _x \in \{0.25, 0.5\}$ and performed 250 draws for each combination. For 17 out of 18 combinations of *n*, $\beta _1$ and $\sigma _x$, we found that $f \in \mathcal {I}$ outperformed $f \in \mathcal {F}$ with respect to median squared error, with an average reduction in median squared error of 54%. For 18 out of 18 combinations, $f \in \mathcal {I}$ outperformed $f \in \mathcal {F}$ with respect to mean squared error, with an average reduction of 51%. For this reason, we chose to set $f \in \mathcal {I}$ as the default option happi and used this class of functions for both our data analyses and error rate simulations.

#### Type 1 error and power simulations

For the type 1 error rate and power simulations shown in the “Simulation study” section, we performed 500 simulations for each combination of $\sigma _x$, $\beta _1$ and *n*. We set a minimum of 16 EM iterations, $t_{\text {max}} = 1000$, $B = 1000$, and $\Delta = 0.1$ for both the null and alternative models.

### Additional files

**Additional file 1.** Supplementary files and figures.

**Additional file 2.** Review history.

## Data Availability

happi is available as an open-source R package under a GPL 3.0 license along with tutorials and workflows at https://github.com/statdivlab/happi and a static version (v0.8.7) has been made available at 10.5281/zenodo.8216120 [42]. The data supporting the conclusions of this article along with code for reproducing our results are made available at https://github.com/statdivlab/happi_supplementary [43] and 10.5281/zenodo.8197577 [44].
