# Supplementary material for: happi: a hierarchical approach to pangenomics inference
Source: Genome Biol. 2023 Sep 29;24:214. doi: 10.1186/s13059-023-03040-6 (PMC10540326; doi:10.1186/s13059-023-03040-6)
Supplement: Supplementary file 1 — Additional file 1. Supplementary files and figures. [file 13059_2023_3040_MOESM1_ESM.pdf]

Additional file 1: supplementary information for happi

Pauline Trinh[1], David S. Clausen[2], and Amy D. Willis[2]

[1]Department of Environmental and Occupational Health Sciences, University of Washington
[2]Department of Biostatistics, University of Washington

August 26, 2023

S1 Algorithm 1: hypothesis testing via permutations

This algorithm describes a hypothesis testing procedure to test $\mathbf{A}\beta = c$ using `happi`. Because the approach is a nonparametric permutation-based testing procedure, we refer to it as `happi-np`. Throughout $\hat{\theta}_0$ denotes the value of the maximum likelihood estimate of the model parameters under $H_0 : \mathbf{A}\beta = c$.

1. Estimate $\hat{\theta}$ and $\hat{\theta}_0$ using (Eq.3) and $\{Y_i, X_i, M_i\}$
2. Compute $Q_{LRT} = 2\left[\mathcal{L}(\hat{\theta}) - \mathcal{L}(\hat{\theta}_0)\right]$ as described in "Hypothesis testing".
3. **for** $b = 1, ..., B$ **do**
   (a) Sample π^* from the set of permutations on n elements. Define $X^{*b} = \pi^*(X)$, that is, X^{*b} is a matrix of the same dimension as $X = \{X_1, X_2, \dots, X_n\}$, but with the columns reordered according to the permutation π^*.
   (b) Estimate $\hat{\theta}^b$ and $\hat{\theta}_0^b$ using Y_i, M_i, X_i^{*b} as in "Hypothesis testing".
   (c) Compute $Q_{LRT}^b = 2\left[\mathcal{L}(\hat{\theta}^b) - \mathcal{L}(\hat{\theta}_0^b)\right]$
4. Calculate the p-value:

$$p = \frac{1}{B+1}\left(1 + \sum_{b=1}^{B} \mathbb{1}\left\{Q_{LRT}^b \geq Q_{LRT}\right\}\right)$$

S2 Sensitivity analysis using *Streptococcus thermophilus* MAGs

To assess the robustness of our results to different assumptions about the level of genome contamination, we conducted a sensitivity analysis of our results to different choices of ε, the probability of observing a gene given that it is truly absent. Our original choice of $\varepsilon = 0.05$ was motivated by the maximum contamination of genomes in our sample being approximately 5%, and for our original analysis we found 219 differentially present genes at the 5% FDR level. We reran our analysis for $\varepsilon \in \{0.01, 0.1\}$. Increasing ε to 0.1 resulted in 187 differentially present genes, while decreasing

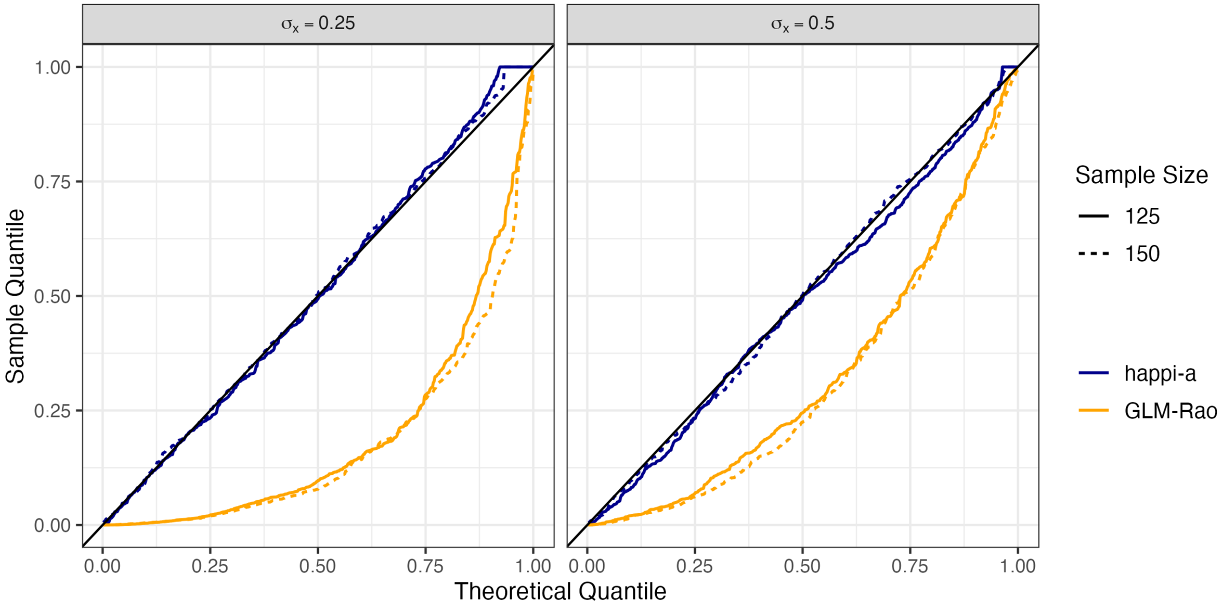

Fig. S1: Motivated by our *S. thermophilus* dataset with $n = 157$, we evaluate the Type 1 error rate control of `happi-a` for large sample sizes and varying degrees of correlation between X_i and M_i. `happi-a` is able to control the Type 1 error rate, behaving near-exactly at larger sample sizes with a rejection rate for a 5% test ranging from 4.9% ($n = 150$ and $\sigma_x = 0.25$; 95% CI: 3.1–6.7%) to 6.8% ($n = 125$ and $\sigma_x = 0.5$; 95% CI:: 4.6–8.9%). In contrast, GLM-Rao exhibits anti-conservativeness at larger sample sizes and all levels of correlation with rejections rates ranging from 20.6% ($n = 125$ and $\sigma_x = 0.5$; 95% CI:: 17.1–24.0%) to 38.3% ($n = 150$ and $\sigma_x = 0.25$; 95% CI: 34.2–42.4%). We show results for 500 simulations. Because `happi-np` is computationally expensive for large sample sizes, it was not feasible to run it for 500 simulations, and therefore we do not show its performance here.

ε to 0.01 resulted in 252 differentially present genes. The smaller number of p-values identified as differentially present by `happi` for larger values of ε is to be expected because differences in gene presence between groups are more attributable to erroneously gene observations. We compare differences in $-\log_{10}$(p-values) for results when using $\varepsilon = 0.05$ compared to $-\log_{10}$(p-values) when decreasing $\varepsilon = 0.01$ and increasing $\varepsilon = 0.1$ in Additional file 1: Fig. S3. Decreasing $\varepsilon = 0.01$ resulted in smaller p-values compared to p-values when using $\varepsilon = 0.05$. Conversely, increasing $\varepsilon = 0.1$ generally increased p-values in comparison to p-values when using $\varepsilon = 0.05$.

S3 Model misspecification simulation study

Here we investigate the Type 1 error rate of `happi` under model misspecification. We specifically focus on the misspecification of $Pr(Y_i = 1|\lambda_i = 1, M_i) = f(M_i)$ as a non-decreasing function in M_i. This is motivated by the concern that in extremely deep short-read sequencing studies, short read assembly tools may fail to assemble fragments of a genome. Thus, the probability of detecting a gene given that it is present may be smaller for high coverages M_i than for moderate coverages. Because obtaining such high coverages for metagenome-assembled genomes is unusual (because it requires extremely deep sequencing or very simple communities), `happi` estimates model parameters under the assumption that $f(M_i)$ is non-decreasing. To investigate the robustness of `happi` to model misspecification in the monotonicity of $f(M_i)$, we generate data with an "inverse-U" relationship between coverage and gene detection, and evaluate `happi`'s error rate control. We

construct a non-monotonic $f(\cdot)$ by fitting a generalized additive model with binomial link function to the observations shown in Fritz et al. 2019 [22] (Figure 3, top left panel, Megahit 1.0.3, 2% error rate curve). The fitted $f(M_i)$ curve that we use for simulation is shown in Additional file 1: Fig. S4. We consider $q = 1$ and $q = 2$, $X_{i1} = 1$, $X_{i2} = \mathcal{N}(\frac{i-1}{n-1}, \sigma = \sigma_x)$, $\varepsilon = 0$ and $\beta = (0, 0)^T$. We evaluate three intervals for the generation of our mean coverages M_i: $M_i = 10 + 90\frac{i-1}{n-1}$, $M_i = 10 + 190\frac{i-1}{n-1}$, and $M_i = 10 + 290\frac{i-1}{n-1}$. By evaluating these three intervals of M_i, we can investigate the effect of increasingly large coverages on `happi`'s error rate control. We simulate data according to the model described in (Eq. 1) and (Eq. 2), and conduct 500 simulations. We ran 500 permutations for `happi-np`. As previously, GLM-LRT and GLM-Rao produced highly similar p-values, and therefore we only show results for GLM-Rao.

The results of our Type 1 error rate simulations are shown in Additional file 1: Fig. S2. In these simulations, all models are misspecified: `happi` is misspecified because of the monotonicity assumption, and GLM-Rao fails to account for differential quality in the genomes. We see that all methods have worse error rate control as the range of coverages increases (top to bottom), and the correlation between X_i and M_i increases (right to left). For low correlation between X_i and M_i ($\sigma_x = 0.5$), `happi-np` and GLM-Rao both appear to control error rates at coverages up to 200X. Both methods have increased error rates as the correlation between X_i and M_i increases ($\sigma_x = 0.25$). All methods suffer when the coverage ranges from 10–300X, which is not surprising as the true probability of detecting a present gene drops to below 30% at $M_i = 300$ (Additional file 1: Fig. S4).

Across $6 \times 500 = 3000$ simulations, the average Type 1 error rate at the 5% level for each method was 13.1% for `happi-a`, 10.5% for `happi-np` and 8.2% for GLM-Rao. Thus, no method's hypothesis tests are robust to model misspecification. Based on our simulation results, `happi` fails to control the Type 1 error rate in more scenarios than GLM-Rao, suggesting that the `happi` is less robust to non-monotonic relationships between quality variables and detection compared to GLM-based approaches. Based on these results, we do not recommend the use of `happi` when analyzing MAGs reconstructed from extremely deeply sequenced short-read data, such as when coverages range up to 300X. However, we also note that GLM approaches may not be reliable in these settings either.

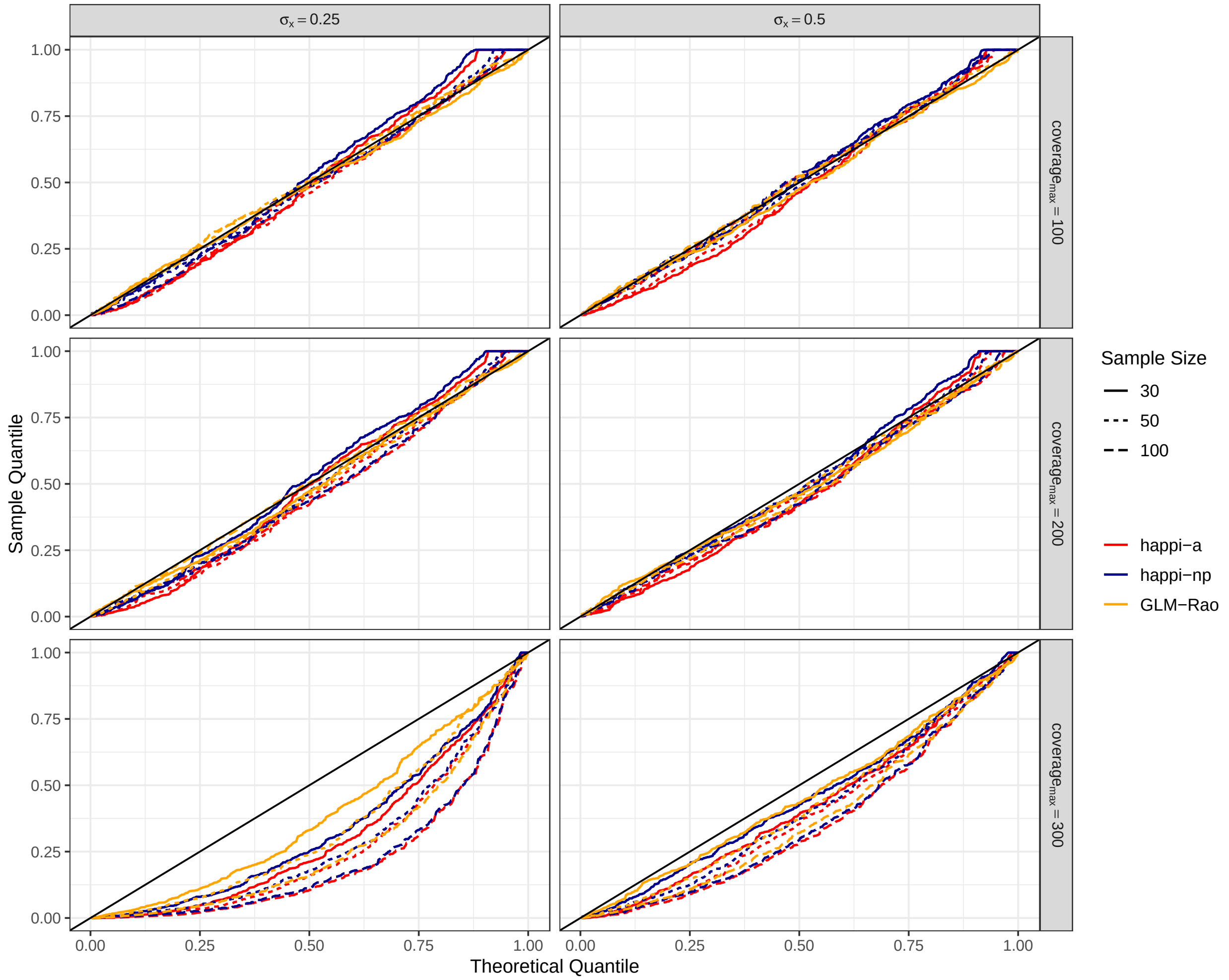

Fig. S2: We evaluate the Type 1 error rate control of `happi-a`, `happi-np`, and GLM-Rao under model misspecification (a non-monotonic relationship between coverage and gene detection). We do not recommend the use of `happi` when analyzing MAGs reconstructed from extremely deeply sequenced short-read data, such as when coverages range up to 300X. However, GLM-Rao may also not be reliable in this setting.

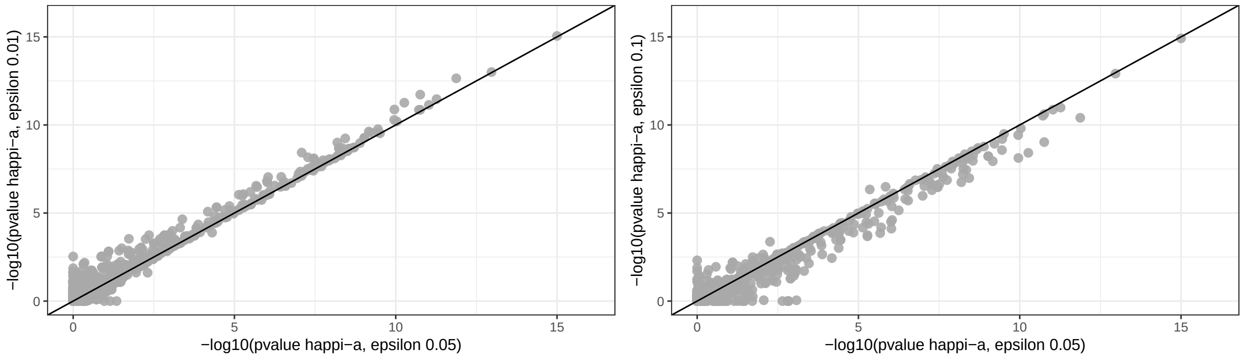

Fig. S3: We investigate the robustness of p-values obtained from running `happi` with different levels of contamination parameter ε. We compare `happi` with $\varepsilon = 0.05$ to `happi` with $\varepsilon = 0.01$ and $\varepsilon = 0.1$. We see that the p-values remain highly correlated across varying values of ε, and the magnitude of very small p-values (top right corner of plots) remains consistent across ε levels. When we decrease $\varepsilon = 0.01$ from $\varepsilon = 0.05$, the p-values tend to become smaller, while when we increase $\varepsilon = 0.1$ from $\varepsilon = 0.05$ the p-values tend to become larger. We discourage further exploration of genes whose significantly differential presence hinges on the assumption of low genome contamination levels, and is not robust across small increases in ε. Data from Richardson et al. 2023 [19].

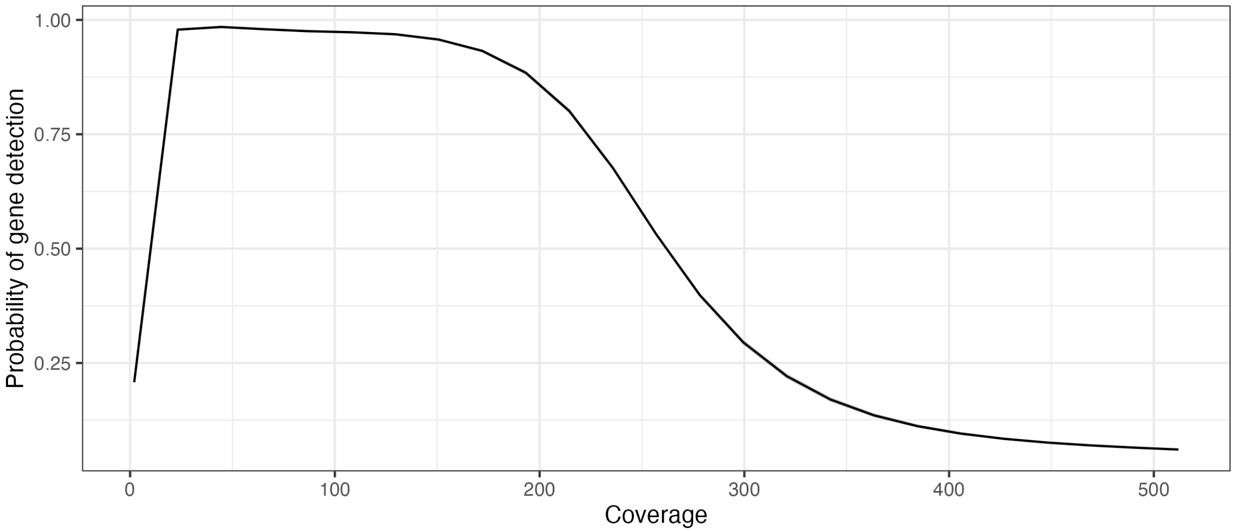

Fig. S4: We estimated the relationship between coverage and the probability of gene detection for high-coverage short read metagenome sequencing studies in order to simulate data with a "inverse-U" relationship between quality variables and detection. Observations from Fritz et al. 2019 [22] (Figure 3, top left panel, 2% error rate curve using Megahit 1.0.3) were smoothed to construct an $f(M_i)$ function.

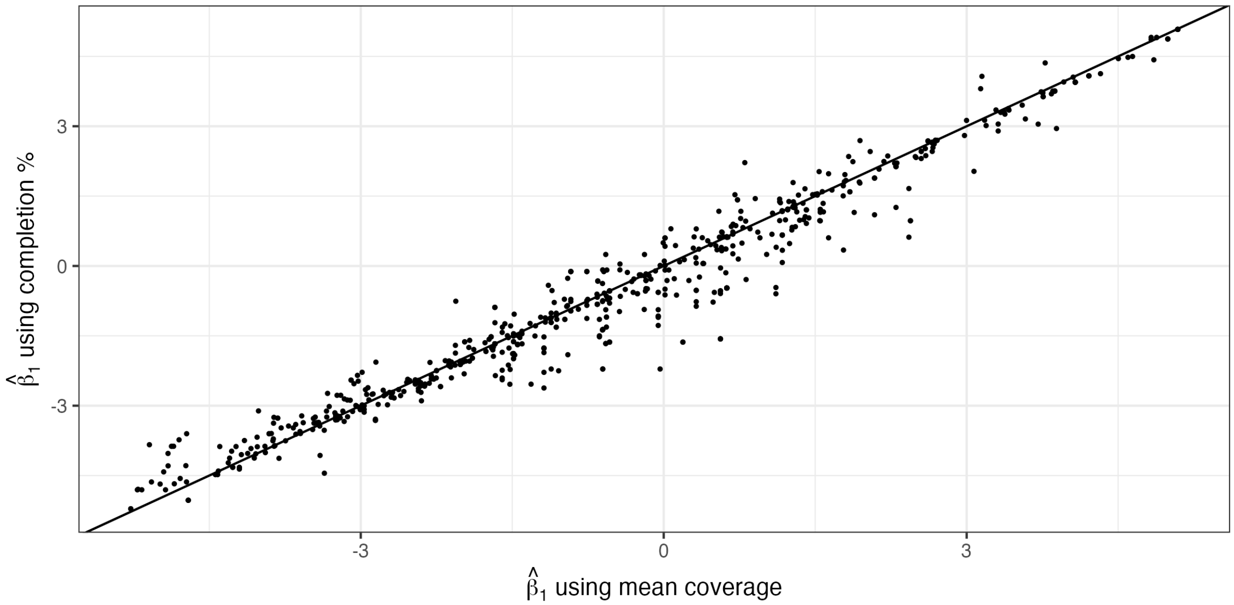

Fig. S5: Different quality variables M_i can be used with `happi`. We compare the β_1 estimates from a model that uses mean genome-level coverage as the quality variable (x-axis) to the β_1 estimates from a model that uses genome completion as the quality variable (y-axis). The β_1 estimates are strongly correlated between the models, suggesting that our results are robust to our choice of M_i. Data from Shaiber et al. 2020 [18]; see section "Data analysis: Saccharibacteria MAGs" for details.
