# Supplementary material for: happi: a hierarchical approach to pangenomics inference
Source: Genome Biol. 2023 Sep 29;24:214. doi: 10.1186/s13059-023-03040-6 (PMC10540326; doi:10.1186/s13059-023-03040-6)
Supplement: Supplementary file 2 — Additional file 2. Review history. [file 13059_2023_3040_MOESM2_ESM.docx]

**1xt round**

**Reviewer 1**

Trinh et al. proposed a novel statistical method happi to perform association analysis between genes and the traits of interests by considering genome quality covariates. happi is designed based on the rationale that genes from the same genomes share similar sequencing depths. The authors proposed an EM algorithm and designed hypothesis testing formula to explore the association signals. The topic is interesting and can potentially substitute the existing gene-based association approaches; several problems should be resolved first.

1. It seems "gene-based" association is outdated and most of the recent MWAS preferred "genome-based" association using the packages such as MetaPhlAn. At least, the authors should prove happi is more efficient than MetaPhlAn in MWAS to convince the users to update their current pipelines.
2. The manuscript does not clearly show how happi can be used in metagenome sequencing data. The experiment used the 43 TM7 MAGs from supragingival plaque and tongue dorsum, which were generated and annotated by another study. Based on my experience, strains can not be fully detected by short-read metagenome assembly from a limited sample size. I think the authors should develop a complete pipeline with the input as the MAGs from binning and apply hippi to a real publicly available metagenome sequencing studies to examine if it can explore the true association signals that can not be detected by the other approaches.
3. In the experiment of TM7 MAGs, happi was compared to GLM-Rao with respect to the p-value and mean coverage. But it is unclear how to determine if the detected and non-detected signals are true. How to select the six proteins? Any biological rationale behind?
4. The data simulation in section 2.5 is based on the equations (1) and (2). Will this simulation setting be unfair to GLM-Rao and GLM-LRT?

Minor points

(1) In line 25 page 4, the likelihood function l is not defined clearly. More information and its complete form should be described.
(2) I noticed the term "hierarchical" appears many times in the paper. Which part of the model is hierarchical specifically?

**Reviewer 2**

In this manuscript, by P. Trinh and colleagues, the authors present happi, a method to detect gene enrichment from MAGs. The basic issue that the authors are trying to solve is that MAGs, even when seen only as "bags of genes," are noisy representations of the true genomes: genes may be both missing (incompleteness) or erroneously detected (contamination). This is an important problem: in fact, in my group, we had already discussed this manuscript — as it was available as a preprint — as we are dealing with this exact issue (and I do not believe that we are unique in this). The statistically approach taken here is very classical, namely using a hierarchical model, whereby the underlying variables (λᵢ in the author's notation) are not directly observed, and only noisy version is observed. The model is fit using EM (expectation-maximization).

Prima facie, the approach is good (although some of the decisions could be brought into question: for example, why is ε a fixed constant?) and the manuscript is very clearly written. The weakness of the manuscript lies in that the validation of the method does not sufficiently convince me of the superiority of the authors approach in practice (after evaluating the method earlier as mentioned, we are currently not using it in our group). Neither the evaluation on the Saccharibacteria MAGs nor the simulated data presented strongly argue for the superiority of this method over others (e.g., the GLM-Rao in anvi'o that the authors use as a baseline).

#1 There is very little detail on the results on Saccharibacteria. We are told that happi identified 171 at FDR 5%, while GLM-Rao identified 175, but we are not told how those overlap at all (if it is simply the case that GLM-Rao reports 4 more genes than happi, this is almost indistinguishable).

#2 Figure 1 is not very informative. The authors chose to compare the p-values, but, most of the time, a difference for larger p-values are simply irrelevant. It is good that neither method reports the ribosomal protein L27 as differentially abundant (it is a single-copy gene in bacteria that is expected to be universally present, see https://www.nature.com/articles/ismej2011189), but the fact that happi has a slightly higher p-value would not lead to different scientific conclusions.

Ideally, the authors would show that their method leads to first to different conclusions and, secondly, that these conclusions should be preferred for some reason or another. By eyeballing, the case of "Preprotein translocase subunit SecE" seems to be a good example where happi reports a p-value that is meaningfully different from GLM-Rao and this seems intuitively correct based on the data shown and the reasoning presented, but one cherry-picked example is not sufficient.

Of course, ground truth is not known, but there are several plausibility checks that could be done. For example, none of the methods should report universal genes are differentially present and genes that code for proteins known to interact should be detected (or not) together. These are only possible suggestions for plausibility analyses.

#3 The choice of dataset to study is very odd in one particular way: although it contains 43 different MAGs, there are only seven different individuals in the study. Therefore, the datapoints are very far from iid. I realize that this is not the point of the present manuscript, but it would still be better to demonstrate the applicability of the method in a more realistic use case.

#4 The simulation study is not very representative. It is not even (contrary to the authors assertion that it "realistically reflects the relationship between coverage and gene detection in shotgun metagenomics studies") a simulation of a metagenome. For example, one important factor that is neglected is that, particularly for metagenomics, recoverability is not a monotonic function of coverage (unlike the author's expectation, see https://arxiv.org/abs/2210.00098 although I feel this is a "known issue" in that I and colleagues have repeatedly observed that there is a sort of inverse-U relationship between coverage and recoverability).

#5 The current approach that we use is to select only MAGs that checkM reports as having >90% completeness and <5% contamination. In principle, if it works, the authors' approach would allow us to use lower quality MAGs and obtain more spower, but it is not clear that this is the case. For example, it appears that there is a single value for the contamination estimate (ε), so we should not be mixing up higher contamination MAGs.

#6 As a curiosity (and as a sanity check for the method), I would be interested in seeing how the authors estimate of f(Mᵢ) correlates with the checkM evaluation of completeness (or other similar tools, such as BUSCO or checkM2). In some sense, they are both estimates of the same quantity and so should be correlated.

**Authors’ response**

Reviewer #1

**Trinh et al. proposed a novel statistical method happi to perform association analysis between genes and the traits of interests by considering genome quality covariates. happi is designed based on the rationale that genes from the same genomes share similar sequencing depths. The authors proposed an EM algorithm and designed hypothesis testing formula to explore the association signals. The topic is interesting and can potentially substitute the existing gene-based association approaches; several problems should be resolved first.**

Thank you for your careful review of our manuscript. We appreciate your feedback and have done our best to address to clarify the aim of our research as well as to improve the manuscript in line with your suggestions. We would like to clarify that happi is not based on the rationale that genes from the same genomes share similar sequencing depths, but instead that shallowly sequenced (meta)genomes are more likely to fail to detect genes. We have attempted to clarify this important point in Section 1: Background (paragraph 3) and Section 3: Discussion (paragraph 2). Thank you for bringing this ambiguity to our attention.

**1. It seems "gene-based" association is outdated and most of the recent MWAS preferred "genome-based" association using the packages such as MetaPhlAn. At least, the authors should prove happi is more efficient than MetaPhlAn in MWAS to convince the users to update their current pipelines.**

Thank you for raising the comparison with models for genome abundance, such as MetaPhlAn. Our goal in developing happi as a model for gene presence is *not* to supplant tools for modeling genome abundance. Instead, it is intended to *complement* genome abundance tools by making a different comparison: comparing the presence of genes in a collection of genomes. We anticipate that many users would run happi alongside MetaPhlAn, allowing them to look at both gene presence alongside genome abundance.  We have clarified this point in the Discussion (paragraph 6).

**2. The manuscript does not clearly show how happi can be used in metagenome sequencing data. The experiment used the 43 TM7 MAGs from supragingival plaque and tongue dorsum, which were generated and annotated by another study. Based on my experience, strains can not be fully detected by short-read metagenome assembly from a limited sample size. I think the authors should develop a complete pipeline with the input as the MAGs from binning and apply happi to a real publicly available metagenome sequencing studies to examine if it can explore the true association signals that can not be detected by the other approaches.**

Thank you for this suggestion. We completely agree that strains cannot be fully detected by short read metagenome assembly from unlimited sample size – this was exactly the motivation for developing a method that accounts for missing genes in comparisons. The happi R-package now includes a vignette demonstrating its use in the R terminal that can be accessed by following the instructions on the package’s GitHub page located at github.com/statdivlab/happi.

We agree that a pipeline is likely to substantially increase the usability of happi. To this end, we have created a workflow showing how a user can perform data analysis with happi starting with a collection of MAGs and a covariate information table and using it to generate a table of p-values corresponding to testing the null hypothesis that genes are equally prevalent across the covariate groups. We have made this workflow available at github.com/statdivlab/happi/workflows with instructions on its usage on the package’s main GitHub page.

**3.  In the experiment of TM7 MAGs, happi was compared to GLM-Rao with respect to the p-value and mean coverage. But it is unclear how to determine if the detected and non-detected signals are true. How to select the six proteins? Any biological rationale behind?**

This is an excellent question; thank you for raising it. Indeed, Reviewer #2 also had this question. For our response, please see our response to Reviewer #2's Question #2.

**4. The data simulation in section 2.5 is based on the equations (1) and (2). Will this simulation setting be unfair to GLM-Rao and GLM-LRT?**

Indeed this simulation is "unfair" to the logistic regression models, because they are misspecified under this data generation structure while happi can accommodate it. However, we argue that this data generation structure is *realistic* (because the f(M_i) curve was generated from subsampling an isolate to see how the probability of detection increases with coverage), and therefore shows the *actual disadvantages* of using a method that does not account for the differential quality of genomes. We have clarified this point in the text in Section 2.6: Simulation Study (paragraphs 1 and 4).

**Minor points**

**(1) In line 25 page 4, the likelihood function l is not defined clearly. More information and its complete form should be described.**

We have now included the full log-likelihood function in equation 6.

**(2) I noticed the term "hierarchical" appears many times in the paper. Which part of the model is hierarchical specifically?**

Statisticians often describe models as hierarchical when the conditional distribution of some (random) variables depends on information about other (random) variables. Our model is hierarchical because the distribution of gene detection depends on gene presence (equation 2: Y_i | \lambda_i, M_i), and the distribution of gene presence depends on the covariates (equation 1: \lambda_i | X_i).

Reviewer #2

**In this manuscript, by P. Trinh and colleagues, the authors present happi, a method to detect gene enrichment from MAGs. The basic issue that the authors are trying to solve is that MAGs, even when seen only as "bags of genes," are noisy representations of the true genomes: genes may be both missing (incompleteness) or erroneously detected (contamination). This is an important problem: in fact, in my group, we had already discussed this manuscript — as it was available as a preprint — as we are dealing with this exact issue (and I do not believe that we are unique in this). The statistically approach taken here is very classical, namely using a hierarchical model, whereby the underlying variables (λᵢ in the author's notation) are not directly observed, and only noisy version is observed. The model is fit using EM (expectation-maximization).**

**Prima facie, the approach is good (although some of the decisions could be brought into question: for example, why is ε a fixed constant?) and the manuscript is very clearly written. The weakness of the manuscript lies in that the validation of the method does not sufficiently convince me of the superiority of the authors approach in practice (after evaluating the method earlier as mentioned, we are currently not using it in our group). Neither the evaluation on the Saccharibacteria MAGs nor the simulated data presented strongly argue for the superiority of this method over others (e.g., the GLM-Rao in anvi'o that the authors use as a baseline).**

Thank you for your review of our manuscript. We appreciate your feedback and insightful questions very much. We hope that the additional results and discussion that we provide below and in the revised text convince you of the superiority of this method over others. Our opinion on the question of superiority is that happi does no worse than GLM-Rao in most realistic scenarios, and can do much better, especially when there is variation in genome quality within a sample. That is, happi provides no-cost insurance against differential data quality. That said, if all genomes are high quality, or if there is zero correlation between genome quality and gene presence, then the two methods perform similarly. However, happi has advantages even if there is weak correlation between genome quality and gene presence, and obviates the need to investigate the correlation between gene quality and (unknown) gene presence.

**#1 There is very little detail on the results on Saccharibacteria. We are told that happi identified 171 at FDR 5%, while GLM-Rao identified 175, but we are not told how those overlap at all (if it is simply the case that GLM-Rao reports 4 more genes than happi, this is almost indistinguishable).**

Thank you for raising this point. We have added additional text to data analysis section 2.4 (paragraphs 3 and 4) describing the differences in results in more detail. Firstly, we note that we have now created two hypothesis testing procedures for happi: a nonparametric permutation-based method best suited for small sample sizes, and a asymptotic approximation best suited for larger sample sizes. As a result, some of our quantitative results differ slightly from the original submission, but none of our substantive results change.

We updated our analyses to utilize happi’s nonparametric hypothesis testing procedure given the modest sample size of n = 47 TM7 genomes. We found that happi identified 176 genes as differentially present when controlling FDR at 5% compared to 219 genes identified by GLM-LRT and 175 genes identified by GLM-Rao. Out of the 176 identified as differentially present by happi, all 176 genes were also identified by GLM-LRT as being differentially present and 166 genes were identified by GLM-Rao as being differentially present. In addition, to address your later point, we have also provided a thorough investigation into the biological plausibility of the Saccharibacteria results, which we hope provides some of the additional details you are interested in. We show that out of 172 core genes tested that belong to the TM7 core genome, happi identified 6 as being differentially present when controlling FDR at 5% compared to 10 core genes identified by GLM-LRT and 7 core genes identified by GLM-Rao.

Taken together, we hope that this illuminates that even if approximately the same number of differentially abundant genes are detected between happi and GLM-Rao, happi gives significant hits that seem more reasonable by deconflating detection and presence while also (slightly) reducing the number of false positives (i.e. significantly differentially present core genes).

**#2 Figure 1 is not very informative. The authors chose to compare the p-values, but, most of the time, a difference for larger p-values are simply irrelevant. It is good that neither method reports the ribosomal protein L27 as differentially abundant (it is a single-copy gene in bacteria that is expected to be universally present, see** [**https://urldefense.com/v3/__https://www.nature.com/articles/ismej2011189__;!!K-Hz7m0Vt54!kPiZg8R1CUTjzbIlVsyIdmNic6jvMINqW12rQuVBctNqwxaHR6HGtEyS6HKbvMthGv0zGeQjj3pKbTI$**](https://urldefense.com/v3/__https:/www.nature.com/articles/ismej2011189__;!!K-Hz7m0Vt54!kPiZg8R1CUTjzbIlVsyIdmNic6jvMINqW12rQuVBctNqwxaHR6HGtEyS6HKbvMthGv0zGeQjj3pKbTI$) **), but the fact that happi has a slightly higher p-value would not lead to different scientific conclusions.**

**Ideally, the authors would show that their method leads to first to different conclusions and, secondly, that these conclusions should be preferred for some reason or another. By eyeballing, the case of "Preprotein translocase subunit SecE" seems to be a good example where happi reports a p-value that is meaningfully different from GLM-Rao and this seems intuitively correct based on the data shown and the reasoning presented, but one cherry-picked example is not sufficient.**

**Of course, ground truth is not known, but there are several plausibility checks that could be done. For example, none of the methods should report universal genes are differentially present and genes that code for proteins known to interact should be detected (or not) together. These are only possible suggestions for plausibility analyses.**

Thank you for these constructive suggestions. Indeed our initial motivation for including  "Preprotein translocase subunit SecE" was (as you say) to give an example where happi gives a more intuitive result than GLM-Rao, but we agree that this does not show a systematic advantage of our proposal. In line with your suggestion, we have

1. included a specific comparison of hypothesis testing for differential abundance of core genes between happi and logistic regression approaches in Section 2.4. We found that happi identifies fewer significantly different core genes than the logistic regression approaches. While the difference is not substantial, we consider this one advantage of happi.
2. shown explicit comparisons of core genes in Figure 1. This includes two cases (Ribosomal proteins S18 and L33) where logistic regression methods would identify a ribosomal protein as significantly differentially abundant (at the alpha=0.05 level) across groups, but where happi does not. In each of these cases, genome quality is conflated with the covariate of interest.
3. included an additional data analysis (response to comment #3). For this larger data analysis, happi identified 3 out of 813 core genes as differentially present when controlling false discovery rate at the 5% level whereas GLM-LRT identified 27 core genes and GLM-Rao identified 6 core genes.

Taken together, we hope that this provides stronger evidence for happi having a lower "false positive" rate (where false positives are defined as significantly differentially abundant core genes) than existing methods, as well as providing more detailed intuitive examples for both significantly differentially abundant genes and not significantly differentially abundant genes (revised Figure 1).

**#3 The choice of dataset to study is very odd in one particular way: although it contains 43 different MAGs, there are only seven different individuals in the study. Therefore, the datapoints are very far from iid. I realize that this is not the point of the present manuscript, but it would still be better to demonstrate the applicability of the method in a more realistic use case.**

Thank you for raising this question. You raise an excellent point about the non-iid nature of the dataset that we originally analyzed. To supplement the manuscript with an additional dataset with iid samples, we have performed an additional data analysis of 157 *Streptococcus thermophilus* MAGs taken from publicly available data from the Unified Human Gastroinestinal Genome (UHGG) collection. An additional advantage of this data analysis is that it has a larger sample size than our original dataset, allowing additional validation.

Details regarding the new data analysis can be found in the revised Section 2.5. The results from our analysis of *S. thermophilus* MAGs show that happi identified 219 differentially present genes when controlling the FDR level to 5% compared to 311 genes by GLM-LRT and 254 genes by GLM-Rao. In terms of validation (as you asked about above), happi identified only 3 differentially present core genes whereas GLM-LRT identified 27 core genes and GLM-Rao identified 6 core genes. (This is the second dataset where happi identified fewer core genes as differentially abundant in comparison to logistic regression-based methods.)

We hope you find this analysis to reflect a more realistic use case based on its independent observations and larger nature. Thank you again for this constructive suggestion.

**#4 The simulation study is not very representative. It is not even (contrary to the authors assertion that it "realistically reflects the relationship between coverage and gene detection in shotgun metagenomics studies") a simulation of a metagenome. For example, one important factor that is neglected is that, particularly for metagenomics, recoverability is not a monotonic function of coverage (unlike the author's expectation, see** [**https://urldefense.com/v3/__https://arxiv.org/abs/2210.00098__;!!K-Hz7m0Vt54!kPiZg8R1CUTjzbIlVsyIdmNic6jvMINqW12rQuVBctNqwxaHR6HGtEyS6HKbvMthGv0zGeQjbfml05w$**](https://urldefense.com/v3/__https:/arxiv.org/abs/2210.00098__;!!K-Hz7m0Vt54!kPiZg8R1CUTjzbIlVsyIdmNic6jvMINqW12rQuVBctNqwxaHR6HGtEyS6HKbvMthGv0zGeQjbfml05w$)  **although I feel this is a "known issue" in that I and colleagues have repeatedly observed that there is a sort of inverse-U relationship between coverage and recoverability).**

Thank you for raising your concerns regarding the data generation process of our simulation study. We are happy to provide further clarification, as well as to investigate the performance of happi under an inverse-U relationship between coverage and probability of gene detection.

We believe that our simulation study is representative of the relationship between coverage and genome quality *in most studies*. We completely agree that for very deep sequencing (very high coverages), short read sequencing combined with short-read assembly tools can lead to poor genome quality. However, the relationship between coverage and probability of detection that we used for simulation was constructed empirically, by subsampling sequenced reads from the sequencing of an Escherichia coli isolate, as referenced in Figure 3. We see a very clear monotonic relationship between coverage and probability of detection in Figure 3. We used this empirically constructed f(M_i) curve in our simulations, which is why we believe that our simulation study realistically reflects the relationship between coverage and gene detection in most shotgun metagenomic studies.

One limitation of our approach to empirically constructing an f(M_i) curve was that we only investigated coverages up to ~25x, as this was approximately the range of coverages in our data analysis (approximately 1-26x). As you note, this may not reflect *all* possible scenarios. Therefore, to investigate the performance of happi in situations where there is a *non-monotonic* relationship between the quality variable and the probability of detecting a gene, we conducted an additional simulation study where we used data exhibiting this inverse-U relationship between coverage and gene detection ([Fritz et al. 2019](https://microbiomejournal.biomedcentral.com/articles/10.1186/s40168-019-0633-6) Figure 3; top left panel; 2% error rate) for our data generation process. Additional simulations to investigate this point can now be found in Appendix Section B. We evaluated three intervals for our mean coverages M_i (where the maximum mean coverage for a given genome i is either 100X, 200X or 300X) to illustrate the impact of including more deeply sequenced genomes in a data analysis. We found that under this data generation procedure (Appendix Figure 4), happi does generally suffer in its rate control, but can control Type 1 error rates when the maximum mean coverage is less than 200X. happi is *less* robust than logistic regression methods under this form of model misspecification, however, logistic regression approaches also do not control error rates when the coverages of many genomes are very high, as we discuss in the revised manuscript.

Our revised manuscript discusses this particular form of model misspecification, and a detailed description of our simulation study can be found in Appendix Section B and Supplementary Figure  5.

Thank you for bringing up this important question. We hope that we have clarified why we believe our simulation is realistic in general (even if it is not realistic for *all* studies, such as extremely high coverage studies). We also hope that our additional simulation clarifies the performance of happi under inverse-U data structures and highlights the level of caution researchers should take when including a large number of very deeply sequenced genomes in their analysis.

**#5 The current approach that we use is to select only MAGs that checkM reports as having >90% completeness and <5% contamination. In principle, if it works, the authors' approach would allow us to use lower quality MAGs and obtain more power, but it is not clear that this is the case. For example, it appears that there is a single value for the contamination estimate (ε), so we should not be mixing up higher contamination MAGs.**

Thank you for bringing up this important point about contamination heterogeneity. While the current implementation of the happi software implements a fixed value of epsilon for each MAG i, we have opened a GitHub issue (<https://github.com/statdivlab/happi/issues/15>) to enhance our software and allow greater flexibility to the user in specifying a given epsilon for each genome/metagenome. We hope to address this issue within the next few weeks, thus allowing the user to provide a genome-specific estimate of contamination.

We completely agree with you that our current manuscript does not provide evidence that including more (but lower quality) MAGs can increase power to detect true differential gene presence. We view this question as a question regarding experimental design: trading off between *quality* and *quantity* of MAGs. We allude to this point in our "future work" section of the Discussion:

*We suggest several avenues for further research. The first is to study the impact of experimental design on the statistical power of our proposed hypothesis testing procedure. Researchers often have to decide how to allocate budget across number of samples (including replicates and control data) and sequencing depth per sample. While existing guidelines for sequencing depth have focused on taxonomy estimation, MAG reconstruction, and gene detection \cite{Royalty2019,zaheer2018impact,quince2017shotgun,Gweon2019,hillmann2018evaluating,sims2014sequencing}, our proposed modeling approach enables the principled study of the design of shotgun sequencing experiments to maximize power to detect differences in gene presence across sample groups.*

We are currently pursuing this line of research and hope to have a more comprehensive demonstration of this point in a future manuscript.

Because we cannot comprehensively address this point in this manuscript, we have performed an additional sensitivity analysis to investigate the robustness of happi's results to the input level of contamination (Appendix Section C and Supplementary Figure 7), finding that it is robust to the choice of epsilon (especially for the smallest p-values). We hope this provides some insights into choosing the level of contamination for a data analysis in practice.

**#6 As a curiosity (and as a sanity check for the method), I would be interested in seeing how the authors estimate of f(Mᵢ) correlates with the checkM evaluation of completeness (or other similar tools, such as BUSCO or checkM2). In some sense, they are both estimates of the same quantity and so should be correlated.**

This is a wonderful suggestion. We compare parameter estimates of beta_1 based on different choices of M_i in Supplementary Figure 8. Using our dataset of TM7 genomes, we investigate using CheckM completion as the quality variable in our model, and compare it to estimates obtained using coverage as the quality variable. Estimates of beta1_hat are highly correlated between both models, suggesting the robustness of the results to the choice of M_i (at least for this particular data set).

We thank both reviewers for their very constructive comments and hope that our revision addresses them fully.

In addition to the above changes to the manuscript, we have also made additional changes to the method and manuscript that we believe improve its clarity and flexibility.

**Additional Manuscript Changes**

1. Removal of Fisher’s exact test

We removed Fisher’s exact test because we believe that the limited use cases of Fisher’s exact test make it an inadequate comparison method with logistic regression and happi.  Specifically, Fisher’s exact test cannot be applied in settings where the covariate is continuous nor can it incorporate multiple covariates when modeling gene presence. For this reason, we cannot evaluate its performance with respect to Type 1 error rate control and power, and therefore hesitate to show its results on our data analysis.

2. Inclusion of a  nonparametric hypothesis testing procedure for more limited sample sizes

As mentioned previously (Reviewer #2, Question #1), we provide an additional nonparametric permutation-based hypothesis testing approach for reliable inference using more modest sample sizes referenced in Section 2.3 and 2.6. We use this approach for the smaller TM7 data analysis, and our original asymptotic approach for the larger *S. thermophilus* dataset.

**2^nd^ round**

**Reviewer 1**

I appreciate your efforts in addressing the comments and issues raised. I am pleased to see that you have made significant improvements to the manuscript. Now the conclusion is well-supported by the evidence you have presented. However, I would like to suggest a few further improvements that could be made to the paper.
1. I noticed a discrepancy between the description in the manuscript and the feedback provided regarding section 2.4 and Figure 1. I wonder if you were comparing happi with logistic regression on the present or differential abundance of core genes. The statement in the discussion suggests that happi is not intended to explore differentially expressed genes.
2. Given that the efficacy of happi is heavily reliant on the quality of MAGs, it would be beneficial to include a simulation study that systematically examines the impact of contamination and coverage. Although I appreciate that the authors have demonstrated the effect of sequencing coverage in Appendix Figure 4, contamination has only been investigated by demonstrating the robustness of the choice of epsilon. As the MAG contamination of Streptococcus thermophilus is still below 5%, Supplementary Figure 7 only serves to prove that happi works well on low-contaminated MAGs. Therefore, I recommend that the authors generate artificial genomes with varying contamination rates (may find the strategy from https://genomebiology.biomedcentral.com/articles/10.1186/s13059-021-02393-0) and discuss the recommended quality thresholds for MAG selection.
3. I strongly recommend that the authors provide detailed guidance on how to apply happi to metagenomic sequencing data in their GitHub repository. This could include step-by-step instructions for users on how to process their sequencing data, from short-read sequencing to genome assembly, contig binning, gene annotation, and the extraction of covariates such as contamination and depth. Such guidance would be especially valuable for users with limited bioinformatics experience who may require additional support in applying happi to their own metagenomic sequencing data.
4. More information should be provided on how to get the data in experiments. E.g. how to select Streptococcus thermophilus MAGs from MGnify? I tried to search Streptococcus thermophilus on MGnify as the keywords and found MGYS00005801 and MGYS00005801, but none of them included Spain and Sweden samples.

**Reviewer 2**

I thank the authors for their work (and apologize for the delay in reviewing
their manuscript). I consider they have answered my comments satisfactorily.

As a very minor phrasing point, is "behaves nearly-exactly" jargon? It reads
awkward as English (one expects "behaves nearly exactly as expected" or
"behaves nearly exactly as predicted by the model"), but perhaps it is a term
of art (page 7, line 20 and Figure 6 caption).

**Authors’ response**

Reviewer #1

**I appreciate your efforts in addressing the comments and issues raised. I**

**am pleased to see that you have made significant improvements to the manuscript. Now**

**the conclusion is well-supported by the evidence you have presented. However, I would**

**like to suggest a few further improvements that could be made to the paper.**

**1. I noticed a discrepancy between the description in the manuscript and the feedback**

**provided regarding section 2.4 and Figure 1. I wonder if you were comparing happi with**

**logistic regression on the present or differential abundance of core genes. The statement**

**in the discussion suggests that happi is not intended to explore differentially expressed**

**genes.**

Thank you for bringing up this discrepancy between the feedback we provided to section 2.4

and the manuscript. As you pointed out, happi is not intended to explore differential abundance

of genes but instead models the presence/absence of genes. We apologize for any confusion

our previous feedback may have raised and have reviewed the manuscript for consistency in

language.

**2. Given that the efficacy of happi is heavily reliant on the quality of MAGs, it would be**

**beneficial to include a simulation study that systematically examines the impact of**

**contamination and coverage. Although I appreciate that the authors have demonstrated**

**the effect of sequencing coverage in Appendix Figure 4, contamination has only been**

**investigated by demonstrating the robustness of the choice of epsilon. As the MAG**

**contamination of Streptococcus thermophilus is still below 5%, Supplementary Figure 7**

**only serves to prove that happi works well on low-contaminated MAGs. Therefore, I**

**recommend that the authors generate artificial genomes with varying contamination**

**rates (may find the strategy from**

**https://urldefense.com/v3/__https://genomebiology.biomedcentral.com/articles/10.1186/s**

**13059-021-02393-0__;!!K-Hz7m0Vt54!hEjBAWhQUb8qutxhqxgjOu1PjiUQU52oEqBkZkv22t**

**MlS1HVEeIHmMdP_bFlAncsZ6HHJF85zxo4Guo$ ) and discuss the recommended quality**

**thresholds for MAG selection.**

Thank you very much for raising this consideration. We agree that this is an interesting and

important open question. However, simulating collections of microbial genomes, developing a

pipeline to automate their post-processing, and investigating the error rates and estimation error

of happi under different levels of contamination make this a substantial request. In discussion

with the Associate Editor, we have decided to leave thresholds for genome quality as an

on-going area of research. Instead, we have added a discussion of contamination (and open

questions in this area) and how it may impact happi's performance in our Discussion section

paragraph 5.

**3. I strongly recommend that the authors provide detailed guidance on how to apply**

**happi to metagenomic sequencing data in their GitHub repository. This could include**

**step-by-step instructions for users on how to process their sequencing data, from**

**short-read sequencing to genome assembly, contig binning, gene annotation, and the**

**extraction of covariates such as contamination and depth. Such guidance would be**

**especially valuable for users with limited bioinformatics experience who may require**

**additional support in applying happi to their own metagenomic sequencing data.**

Thank you for this suggestion. In response, we have greatly expanded happi's documentation

so that users may better understand where happi gets implemented in a metagenomics

workflow (after assembly, binning, refinement, annotation) and have linked users to a high-level

overview of tools used in various steps of bioinformatics pipelines for processing shotgun

metagenomics data. While we provide possible options, we leave the choice of specific tools for

conducting each step of pre-processing to the user. We additionally link users to tutorials from

the anvi’o platform that wraps together all the necessary steps for assembly, binning,

refinement, and annotation.

**4. More information should be provided on how to get the data in experiments. E.g. how**

**to select Streptococcus thermophilus MAGs from MGnify? I tried to search**

**Streptococcus thermophilus on MGnify as the keywords and found MGYS00005801 and**

**MGYS00005801, but none of them included Spain and Sweden samples.**

Thank you for raising this. We have provided further detail on how these data can be accessed

using MGnify in our Methods: Streptococcus thermophilus Section 4.2. Additionally, the data is

also available on our GitHub repo at

https://github.com/statdivlab/happi_supplementary/tree/main/data.

Reviewer #2

**I thank the authors for their work (and apologize for the delay in reviewing**

**their manuscript). I consider they have answered my comments satisfactorily.**

**As a very minor phrasing point, is "behaves nearly-exactly" jargon? It reads**

**awkward as English (one expects "behaves nearly exactly as expected" or**

**"behaves nearly exactly as predicted by the model"), but perhaps it is a term**

**of art (page 7, line 20 and Figure 6 caption).**

Thank you for raising this – it is a great question, and we agree that the terminology is

field-specific.

Hypothesis tests can be described as anti-conservative (rejecting a true null hypothesis too

often), exact (rejecting a true null hypothesis exactly 100*alpha% of the time), or conservative

(rejecting a true null hypothesis less often). Therefore, when we say near-exact, we are referring

to the fact that happi's rejection rate for a 5% test ranges from 4.9% (n=150 and sigma=0.25;

95%CI: 3.1-6.7%) to 6.8% (n=125 and sigma=0.5; 95%CI: 4.6-8.9%) which is close to the

nominal level ("near exact").

Thank you again for this question. We have explained this terminology at its first appearance, in

Section 2.6.
